# Rearing and Shipping of *Uranotaenia lowii*, a Frog-Biting Mosquito

**DOI:** 10.21769/BioProtoc.4996

**Published:** 2024-06-05

**Authors:** Richa Singh, Neil D. Sanscrainte, Alden S. Estep, K. González, Ximena E. Bernal

**Affiliations:** 1 Department of Biological Sciences, Purdue University, West Lafayette, IN, USA; 2 Fly and Mosquito Research Unit, Center for Medical, Agricultural & Veterinary Entomology, Agricultura Research Service, USDA, Gainesville, FL, USA; 3 Smithsonian Tropical Research Institute, Panamá, República de Panamá

**Keywords:** Non-human host-biting mosquitoes, Egg, Eavesdropper, Larva, Pupa, Adult, Colony, Blood feeding, Uranotaeniini, *Uranotaenia lowii*, Husbandry

## Abstract

Many studies on mosquito biology rely on laboratory-reared colonies, emphasizing the need for standardized protocols to investigate critical aspects such as disease biology, mosquito behavior, and vector control methods. While much knowledge is derived from anthropophilic species from genera like *Anopheles, Aedes*, and *Culex*, there is a growing interest in studying mosquitoes that feed on non-human hosts. This interest stems from the desire to gain a deeper understanding of the evolution of diverse host range use and host specificity. However, there is currently a limited number of comprehensive protocols for studying such species. Considering this gap, we present a protocol for rearing *Uranotaenia lowii*, a mosquito species specialized in feeding on anuran amphibians by eavesdropping on host-emitted sound cues. Additionally, we provide instructions for successfully shipping live specimens to promote research on this species and similar ones. This protocol helps fill the current gap in comprehensive guidelines for rearing and maintaining colonies of anuran host–biting mosquitoes. It serves as a valuable resource for researchers seeking to establish colonies of mosquito species from the Uranotaeniini tribe. Ultimately, this protocol may facilitate research on the evolutionary ecology of Culicidae, as this family has recently been proposed to have originated from a frog-feeding ancestor.

Key features

• Rearing and maintenance of colonies of non-human host-biting mosquitoes that feed on frogs using host-emitted acoustic cues.

• Provides shipping guidelines aimed to enhance the establishment of colonies by new research groups and specimen exchanges between labs.

## Background

An essential component in the comprehensive study of mosquitoes involves sustaining laboratory populations that allow experimental manipulations. Mosquito colonization enables in-depth exploration of life history traits, behavior, and the physiological mechanisms underlying them. Colonization of mosquitoes of medical importance, such as *Anopheles gambiae, Aedes aegypti*, and *Culex pipiens*, has, for instance, accelerated the rate of understanding of the biology of these anthropophilic species [1–4]. Establishing and maintaining robust and standardized mosquito colonies requires the development and application of well-established protocols as well as a good understanding of the key factors influencing mosquito fitness in captivity. Recent interest in mosquitoes that feed on non-human hosts has increased due to the increased recognition of the implications that mosquitoes feeding on other hosts can have in the ecosystem [5,6]. Until now, however, little was known regarding the rearing techniques and successful establishment of laboratory populations of mosquitoes with non-human hosts. Among those, mosquitoes that feed on frogs provide a valuable opportunity to understand the evolutionary ecology of Culicidae, as this family has recently been proposed to have originated from a frog-feeding ancestor [7]. While some mosquitoes are generalists and opportunistically feed on frogs as well as other hosts, other mosquitoes are specialized in feeding exclusively on anurans. The use of host-emitted acoustic signals, such as the mating calls of frogs for instance, is an adaptation that has evolved independently multiple times across flies and mosquitoes to detect and localize anuran hosts [8]. Among such frog-biting mosquitoes, *Uranotaenia lowii* is characterized by its preference for anuran hosts [9] and the use of auditory cues to locate them [10,11]. To date, no detailed documented studies have outlined protocols for maintaining laboratory colonies for *Ur. lowii* (but see Chapman [12]) or any other frog-biting mosquito species. Here, we present a standardized protocol for the rearing and colony maintenance of *Ur. lowii* mosquitoes. We also include a protocol for effectively shipping *Ur. lowii* specimens to promote specimen exchange between research groups.

## Materials and reagents

**Biological materials**

**Hosts for blood feeding**

*Uranotaenia lowii* feed from anuran hosts in nature [9]. In contrast to mosquito species from genera like *Aedes* and *Anopheles*, which can often be raised using artificial blood diets [13], it is recommended to feed frog blood for the rearing of *Ur. lowii*. Field host surveys show that this species feeds on frogs from different families [9], so a variety of anuran species are likely to support a colony of *Ur. lowii*. We recommend, however, the following hosts to be used for blood feeding, as they successfully promote robust egg production: barking tree frog (*Dryophytes gratiosus*), Cuban tree frog (*Osteopilus septentrionalis*), cane toad (*Rhinella marina*), and southern toad *(Anaxyrus terrestris*) [12,11]. These anuran species can be procured from local shops or online stores (e.g., https://joshsfrogs.com/ or https://www.backwaterreptiles.com/).

*Note: Protocols for using anurans for blood feeding must be approved by the appropriate Institutional Animal Care and Use Committee.*

***Uranotaenia lowii***

This protocol describes the rearing and maintenance of *Ur. lowii* strain MFRU-FL (NCBI BioSample: SAMN33601576). It is to be expected, however, that these guidelines are transferable to other strains of this species. This protocol also provides a foundation for developing similar approaches to maintaining colonies of other *Uranotaenia* frog-biting species (i.e., de Silva et al. [14]). The colonies were originally established from egg rafts obtained from the USDA-ARS-CMAVE Mosquito and Fly Research Unit (Gainesville, FL, USA).

**Reagents**

1. Deionized water
2. Potbelly pig chow powder finely sieved (i.e., Mazuri^®^ Mini Pig Youth); grind using a porcelain mortar and pestle (Letoyi, catalog number: B08LB3TJL4) and sieve through a fine mesh strainer (Clscea, catalog number: B0CBDFW92V)
3. Bovine liver powder (i.e., MP Biomedicals, catalog number: 290039601)
4. Brewer’s yeast (i.e., MP Biomedicals, catalog number: 290331205)
5. White sugar (e.g., Domino Cane Granulated Sugar, catalog number: 83FF)
6. Bleach (Clorox, catalog number: 30966, concentrated regular bleach)
7. 70% ethanol (sterile 70% denatured ethanol) (Texwipe, catalog number: TX3265)

**Solutions**

1. Liver powder suspension (larval food) (see Recipes)
2. Sucrose solution [10% (w/v) adult food] (see Recipes)

**Recipes**

1. **Liver powder suspension (larval food)**
  **Table:** 

Reagents	Quantity or Volume
Bovine liver powder	3 g
Brewer’s yeast	2 g
Deionized H_2_O	100 mL
*Note: Shake well before each use. Can be stored at 4 °C for a week.*
2. **Sucrose solution (adult food)**
  **Table:** 

Reagents	Quantity or Volume
White sugar (e.g., Domino Cane Granulated Sugar)	10 g
Deionized H_2_O	100 mL
*Note: Mix the reagents well before use.*

**Laboratory supplies**

While we provide manufacturers’ names and models for some items, we are not endorsing or promoting those specific products. Instead, we offer them as examples and, when possible, suggest low-cost alternatives to facilitate accessibility for a wider range of laboratories.

1. Cotton balls, size: 5 cm, prepared using any cotton roll (e.g., Intrinsics, catalog number: 227200)
2. Transfer pipettes [Fisherbrand Transfer Pipette, 4.6 mL (Fisher Scientific, catalog number: 13-711-274MD)], or wood applicator sticks (i.e., Fisher Scientific, catalog number: 22-363-158), or plastic spoons obtained from stores for transferring egg rafts
3. Scissors (8-inch titanium scissors) (Westcott, catalog number: 13901)
4. Brown, non-white, seed germination paper (38-lb regular weight creped seed germination paper) (Anchor Paper Company, catalog number: NC1466201). Prepare strips of 17.5 cm length × 8 cm width using scissors
5. Labeling tape (Write-On label tape, white, 1/2" roll) (Research Products International Corp, catalog number: 560172)
6. Manual insect aspirator (Forestry Suppliers, catalog number: 53758)
7. Digital thermometer and hygrometer (i.e., ThermoPro TP49 Digital Hygrometer; temperature range: 14 °C–70 °C; humidity range: 10%–99%)
8. Oviposition and emergence cups: plastic cup (Rubbermaid 2-Cup, catalog number: 7J60)
9. Larval rearing trays; plastic trays, size: 38 cm length × 30 cm width × 7 cm depth (any polypropylene plastic tray can be used, e.g., United Scientific Supplies, catalog number: 81702)
10. Shipping supplies
  - Cardboard box (small to medium to fit the Styrofoam box; Uline outer shipping carton with insulated Foam Shipping Kit, catalog number: S-12682)
  - Styrofoam box with a lid (Uline Insulated Foam Shipping Kit, catalog number: S-12682)
  - Bubble wrap (Uline Air Bubble Wrap Roll, catalog number: S-5120)
  - Small Petri dishes (i.e., one 75 mm diameter petri dish can contain around 10 egg rafts) (non-sterile transparent Polypropylene Petri dish: United States Plastic Corp., catalog number: 89824)
  - Germination paper cutout in circles (diameter determined by Petri dish diameter) (Anchor Paper Company, 38 lb regular weight creped seed germination paper, catalog number: NC1466201)
  - Ice packs (two minimum) (Uline cold packs, catalog number: S-7890)
  - Industrial tape 2 Mil (Uline, catalog number: S-423)

## Equipment

1. Environmental chamber
  The use of environmentally controlled chambers (insectaries) dedicated to mosquito rearing is encouraged (e.g., Percival WE-35VL, Boone, IA). However, it is possible to maintain the colony in a small room in which temperature and humidity are maintained close to target values (temperature: 22–28 °C, humidity 50%–80%, 12:12 h light/dark photoperiod).
*Note: If there is no environmental chamber, the relative humidity within the cage can be increased by placing a humidifier (e.g., Vick humidifier, catalog number: V745A/V745-JUV; not restricted to any specific manufacturer) near the rearing cage and covering the entire setup with a plastic shelf cover (e.g., Formosa covers premium wire shelf cover, heavy-duty storage solution for wire shelving rack, #shelf 4919 pvc off white). The humidifier must be regularly filled with deionized water to regulate the relative humidity in the enclosure.*
2. Frog restraining cage
  A cylindrical cage (height: 8 cm; diameter 12 cm) made entirely of mesh. The top mesh can be replaced by an acrylic circular sheet (i.e., PET Thick Plexiglass sheet; 3 mm, Uline Acrylic sheet, catalog number: S-22486) to observe blood-feeding behavior during experiments. The frog restraining cage should be made of a robust mesh that has holes big enough (1 cm) for the mosquitoes to enter but not too large for the frog to escape (e.g., Home Intuition Heavy Duty Plastic Gutter Guards Mesh, catalog number: B07D91Y8BC).
*Note: The use of a frog restraining cage is essential for limiting the movement of the frogs to increase the likelihood of blood feeding. Limited host movement promotes feeding opportunities and reduces frog defensive behaviors such as swatting, a common strategy used by anurans to repel attacking mosquitoes and midges (i.e., de Silva et al. [15]). Additionally, restraining the frogs prevents them from consuming mosquitoes. It is also advisable to provide a substantial meal for the frog before placing it in the restraining cage, so they are less motivated to eat the mosquitoes.*
3. Mosquito rearing cage
  Mosquito cages that provide access through a sleeve (e.g., BioQuip lightweight aluminum collapsible cages; 46 cm × 46 cm × 46 cm; 2 mm × 2 mm mesh size; Mosquito Rearing Cage, catalog number: 4S4545).

## Procedure

- **Environmental conditions for rearing *Ur. lowii***
  Rear *Ur. lowii* (sex ratio 1:1) in an insect cage inside an insectary room with controlled environmental conditions (temperature: 22–28 °C, humidity 50%–80%, 12:12 h light/dark photoperiod). Mosquitoes require constant access to moisture, and they perform best in humid environments.
- **Blood feeding and oviposition**
  1. Egg production starts with adult females blood feeding on a frog. As in other species of mosquitoes, only female mosquitoes feed on blood, which provides the nutrients necessary for egg development. To promote feeding, place an adult frog in a frog restraining cage inside the rearing cage for 3 h (Figure 1A).
  2. Place oviposition cups half-filled with deionized water (250 mL) and semi-submerged strips of brown seed germination paper for egg laying inside the mosquito cage immediately after removing the frog (Figure 1B). Females take two days to start laying eggs after feeding on their anuran host.
*Note: When placing the frog in the restraining cage, it is important to include moist paper towels at the base to create a humid and comfortable environment for the frog. After 3 h of feeding, remove the frog from the restraining cage and place them back in the housing tank. Additionally, ensure abundant food and water are provided after each use to support their well-being. To reduce stress on the frogs, refrain from using the same individual repeatedly, and instead rotate the use of different individual frogs. Increasing the time between feedings using the same individual frogs as much as possible is advisable. Allowing a recovery time of a month for the same individual frog has worked well in promoting good health. For routine maintenance, mosquito colonies should be fed with blood every other week. Before blood feeding, remove the cotton balls containing the 10% sucrose solution. The age at which* Ur. lowii *mosquitoes first blood feed has not been investigated. Based on our observations, female* Ur. lowii *mosquitoes successfully feed from a frog after one week of age. Hence, we propose this timeframe for the initial blood meal offered to adult mosquitoes.*
  **Figure 1.**
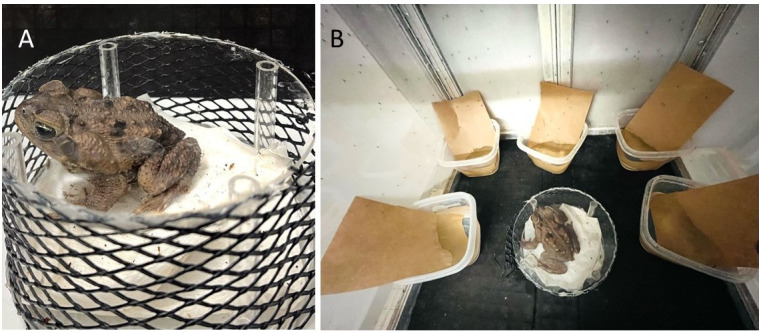
Blood feeding set up for *Ur. lowii* colony. A. Cane toad placed in a frog restraining cage built with mesh and topped with a transparent plexiglass lid. B. Frog restraining cage placed inside the mosquito rearing cage with adult *Ur. lowii*. Oviposition cups half-filled with water and brown germination paper are placed along the walls of the rearing cage. No cotton balls containing sucrose solution are present to promote frog-feeding behavior.
- **Egg collection and hatching**
  1. Like other species from the subfamily Culicinae, *Ur. lowii* also produces egg rafts [16]. Blood-engorged females lay egg rafts in oviposition cups, which should then be moved using a transfer pipette to rearing trays filled with 1.5 L of deionized water (water temperature 20–26 °C) (Figure 2A). To facilitate the uptake of the egg rafts without damaging them, we suggest using a transfer pipette after cutting the tip to increase the opening (5 mm wide) and a plastic spoon or wood applicator sticks to gently lift the rafts out of the water without breaking apart.
  2. Once the egg rafts are transferred to the larval trays, add a pinch (0.16 g) of potbelly pig chow powder finely sieved to promote hatching. Label the tray with the hatching date, experiment type, or other relevant information. A maximum of 10–15 egg rafts should be placed in each tray marked at the level equivalent to 1.5 L to avoid overcrowding.
  3. Place fresh oviposition cups half filled with deionized water daily in the adult mosquito cage to promote oviposition of new egg rafts. After exposure to a frog in the restraining cage, females lay eggs for approximately a week, so regular monitoring and transferring of egg rafts into rearing trays is repeated daily until no additional rafts are laid.
*Note: Egg rafts of* Ur. lowii *are oviposited directly into the water [17] and die if they desiccate. Unlike the eggs of other mosquitoes, such as* Ae. aegypti *whose eggs can be stored for several months [3], the egg rafts of* Ur. lowii *cannot be stored long term. Hatching can be delayed by 1–2 days by storage at 4–8 °C, but their viability under these conditions rapidly decreases.*
  **Figure 2.**
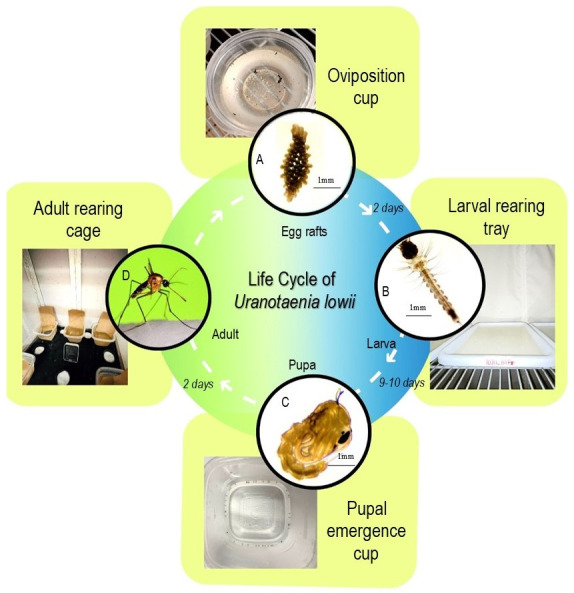
Life cycle of *Ur. lowii* and colony care at each developmental stage. A. Egg raft stage: rafts are laid inside oviposition cups half-filled with deionized water. B. Larval stage: larval development involves four instar stages that take place in the rearing trays with deionized water feed with liver powder suspension (see details under larval rearing). C. Pupal stage: manually picked pupae placed in half-filled emergence cups. D. Adult stage: adult rearing cage equipped with half-filled water cups with germination paper, adult food in cotton balls containing 10% sucrose solution and a digital thermometer and hygrometer to record environmental conditions.
- **Larval rearing**
  1. Egg rafts take two days to hatch into larvae in a rearing tray filled with 1.5 L deionized water when maintained at a density of 1.3–2 larvae per cm^2^ of surface area at approximately 20 °C water temperature. Larvae can be counted manually. Maintain the water level for each tray during the entire rearing process.
  2. Provide larval food as liver powder suspension every other day. Feed each tray with 30 mL of liver powder suspension (Figure 2B). Feed the larvae with a transfer pipette by placing the food on the bottom of the tray, then mix the food well until the water turns light brown. Ensure that the food is evenly distributed across the tray.
*Note: Water in rearing trays might dry out, so it is necessary to refill the tray with deionized water until reaching the mark of 1.5 L. When feeding the larvae, check the color and scent of the water in the trays. If the water appears dark brown, emits a strong odor, or if dead larvae are noticed near the bottom, it is advisable to promptly move the mosquito larvae to a clean container filled with clean deionized water and recently mixed food.*
- **Pupation**
  1. Upon reaching the fourth instar stage, larvae take approximately three days to pupate. Pick pupae manually from the larvae trays using a transferring pipette (cut off the tip by 2 cm to increase the opening so it is 5 mm wide) to move them into half-filled emergence cups.
  2. Place the emergence cups back into the adult cage (Figure 2C). No food is to be provided as pupae do not feed. Repeat the above procedure daily for pupae collection until all larvae have reached this developmental stage.
*Note: It is necessary to regularly remove the pupae from the larval trays and place them into an adult cage at least once a day to ensure that no recently emerged adult mosquitoes escape. In our experience, the pupal phase is the most sensitive period in* Ur. lowii *development. Therefore, it is also important to ensure that the cups containing pupae are not overcrowded. It is recommended to put 50 pupae per cup.*
- **Adult**
  1. Pupae emerge into adults after two days in the pupal stage. Provide constant access to adult food of 10% (w/v) sucrose solution in cotton balls and water wicks in the adult insect rearing cage (Figure 2D). To make sure mosquitoes can land safely to feed without being trapped by loose threads, compact the cotton by rolling each ball with the palm of your hands before soaking them with sugar solution (10% g/mL or one tablespoon of sugar in 100 mL of deionized water).
  2. Place the containers with pupae inside the rearing cage in an evenly distributed pattern to make sure food is easily accessible for all individuals. Replace food and water on alternate days to prevent mold growth or fermentation of the sugary solution. While changing food and water, mosquitoes may try to escape, so shake the containers checking that there are no adults perched on them before removing the containers from the cage. Use a manual aspirator to catch any mosquitoes that may try to escape and place them back in the cage.
*Note: Both male and female mosquitoes need access to water and sugar sources for reproduction and survival. Although adult mosquitoes require substantially less careful monitoring than the larval and pupal phases, it is necessary to perform daily checks of the cages containing adult mosquitoes to ensure that they have continuous access to the sugary solution and water.*
- **Maintenance**
  1. Maintain and regularly monitor the required temperature, humidity, and light conditions. We recommend daily checks of the condition of the colony.
  2. If humidifiers are used, they should be filled with deionized water as often as necessary to maintain the target relative humidity relatively constant. A 1.5-gallon humidifier (see Equipment for details) needs to be filled daily.
  3. Ensuring proper larval and adult feeding can help in maintaining healthy colonies. Blood feeding every other week can lead to longer lifespans of mosquitoes. To avoid overcrowding, it is recommended to have a colony size of around 2,000 mosquitoes in the aforementioned rearing cage.
  4. Clean and disinfect the frog restraining cage, emergence and oviposition cups, aspirator collection tubes, etc., by rinsing them in hot water or soaking them for at least 15 min in 10% bleach after every use. Rinse thoroughly and avoid using soap.
  5. Regularly maintain mosquito cages by replacing the deionized water and cotton balls containing sucrose solution and aspirating dead mosquitoes every other day. Clean cages with 70% ethanol every six months.
  6. The eggs of *Ur. lowii* mosquitoes cannot be stored. To dispose of eggs, leave them overnight in a cup with 10% bleach before discarding them into the sink. Similarly, excess larvae or pupae can be transferred to a container with 10% bleach. Check the container for larvae before discarding it into the sink and run hot water for several minutes afterward. The excess adults should be kept in a freezer in a container overnight before disposal.
- **Shipping egg rafts**
  The egg raft stage is recommended for shipping, as adults are prone to higher mortality during shipping conditions. As elevated mortality has been observed during the larval stage in laboratory conditions, we discourage shipping specimens at this stage.
  1. Preparation
    In a small plastic Petri dish (diameter: 75 mm), up to 10 egg rafts can be shipped on germination paper. First, prepare by cutting the germination paper into approximately 50 small circles of 75 mm in diameter using scissors, so they fit tightly within the Petri dish. To create a depression that is safe for the egg rafts to travel, use these circular germination papers to have two types: (i) unmodified circles, and (ii) donut-shaped circles where there is a single, central small hole (20 mm diameter) in each piece. At the bottom of the Petri dish, add a base layer of unmodified circles of germination paper until it reaches approximately 1/3 of the height of the dish. Then, place donut-type germination paper circles to create a depression that is approximately 10 mm deep. Add deionized water to completely dampen the germination paper and create a shallow water pool for the egg rafts (Figure 3). Use a transfer pipette or a wooden applicator stick to carefully transfer up to 10 egg rafts, so they are floating on the water. Check that the egg rafts are not upside down when placing them in the pool. Once the egg rafts are placed, add one unmodified circle of germination paper to cover the egg rafts.
*Note: Plan to feed the colony 3–4 days before the expected shipment day, so you can obtain egg rafts as close to that day as possible. Shipping should occur as soon as the mosquitoes lay the eggs to avoid hatching during shipment. Consider the weather during shipping, as extreme, low, or high temperatures can expose the package to deadly conditions for the mosquitoes. When possible, shipping the mosquitoes during the spring and fall when conditions are mild should be favored. It is important to note that the volume of water for the egg rafts should be large enough to allow the egg rafts to float on the surface but not too high to result in turbulence that can displace the egg rafts out of the pool where they can desiccate.*
  2. Packing, shipping, and receiving egg rafts
    Place the Petri dish lid and, using tape, secure it to the bottom, sealing it tightly to minimize leaking. Once the Petri dish is ready, place it in the middle of a Styrofoam box and add ice packs to compress the Petri dish(es) (Figure 3). Fill the space of the box with bubble wrap to avoid movement of the Petri dish inside the box during transportation. The Styrofoam should be tightly closed, securing the lid with abundant tape and placed in a cardboard box. Before shipping, add large visible arrows indicating which side goes up and place the shipping label. Since the eggs take 48 hours to mature, shipping overnight is recommended.
    **Figure 3.**
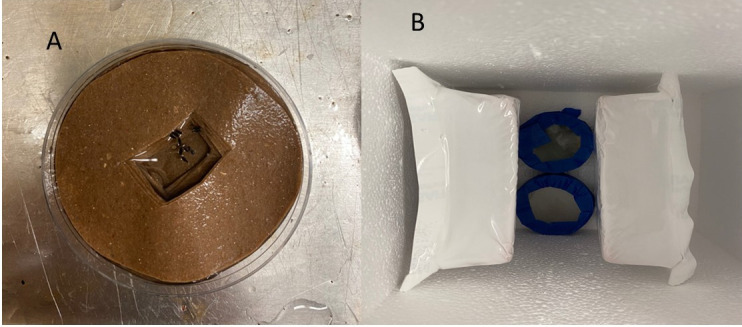
Packing of egg rafts for shipping. A. Egg rafts floating in the water pool made in the germination paper. B. Sealed Petri dishes with egg rafts inside placed in a Styrofoam box equipped with ice packs. Fill empty places with bubble wrap for shipping.
    On the receiving end, carefully take out the Petri dishes and transfer the eggs to larval trays by gently moving the germination paper into the tray and adding deionized water, so the eggs continue to float as the water level increases. The germination paper can be removed from the trays once the water level has elevated the egg rafts.

## Data analysis

This protocol does not directly involve data analysis, but it is the foundation for producing specimens that allow the implementation of diverse experimental approaches to address questions using frog-biting mosquitoes. For example, this protocol facilitated the data acquisition for neurophysiological and behavioral experiments conducted on mosquitoes reared using this method [11].

## Validation of protocol

This protocol has been validated by implementing it in four different laboratories across different institutions: 1) Fly and Mosquito Research Unit, Center for Medical, Agricultural & Veterinary Entomology, Agricultura Research Service, USDA, Gainesville, FL 32608; 2) Laboratory of Neurogenetics and Behavior, HHMI – The Rockefeller University, New York, NY 10065; 3) Texas Tech University, 2500 Broadway W, Lubbock, TX 79409; 4) Sensory and Behavioral Ecology Lab, Purdue University, West Lafayette, IN 47907. During this process, it was validated using different anuran hosts and improved and refined to produce this cost and time-efficient approach to rearing a sustainable colony of frog-biting mosquitoes [11].
